# War, higher education and development: the experience for educationalists at Gaza’s universities

**DOI:** 10.1007/s10734-024-01353-4

**Published:** 2024-11-16

**Authors:** Mona Jebril

**Affiliations:** 1 Centre for Business Research, University of Cambridge, Cambridge, UK; 2 Present Address: Queens’ College, University of Cambridge, Cambridge, UK

**Keywords:** War, Gaza Strip, Higher education, Experience, Oppression, Symbolic violence

## Abstract

The 2014 Israeli war on the Gaza Strip was described, as the ‘longest’ and ‘most violent’, compared to previous wars since 2008. This paper reports on this war experiences for educationalists (academic staff and students) at two of Gaza’s universities. It draws on 36 in-depth semi-structured interviews with educationalists in the Gaza Strip, which I conducted via Skype and mobile/phones from the UK, for my PhD research at the University of Cambridge. Theoretically, the inductive study uses insights from Freire’s *Pedagogy of the Oppressed* and Bourdieu’s work on symbolic violence, retrospectively. Findings from this research show the impact of the war on Gaza’s educationalists has varied between vulnerability and resilience. The memories of loss, fear, and dehumanization continued to affect educationalists, even after the war came to halt. Some of Gaza’s universities buildings, and facilities were also damaged. Consequently, Gaza’s universities found themselves in a dilemma on how to manage immediate needs, with developmental prospects. This paper documents the history of Gaza’s universities, enhancing our sociological understanding of the experiences of higher education in the Gaza Strip, and the challenges for its development. The research widens the geographical scope of research on conflict, and education, by including the experiences of educationalists in the occupied and besieged context of Gaza, which is significantly under-researched. Insights from this research could be useful to inform the process of reconstruction of higher education in the Gaza Strip, after Israel’s ongoing war on the Gaza Strip, since 7th October 2023 comes to end.

## Introduction

This paper explores the experiences of educationalists at Gaza’s universities in the 2014 Israeli war on the Gaza Strip. Bouris (2015) explains that, codenamed by Israeli military as ‘Operation Protective Edge’, the 2014 war on the Gaza Strip, “was the third” since only 2008 (p.111). That said, violence in the Gaza Strip is not coincidental; it is recurrent and impacting on all aspects of its life, including its universities and educationalists. The term ‘educationalists’ refers to students and academic staff at the Faculties of Education at two of Gaza’s universities. Combining both under the term ‘educationalists’ is an attempt to depart from the old examination-oriented practices of creating a chasm between staff and students; nonetheless, in the Gaza Strip context, which is a traditional Arab context of Higher Education (HE), it is a fact that there is a chasm by hierarchy of role and age, which may reflect on their experiences. Echoing this contrasting situation, the article uses both ways of referencing interchangeably.

After the 7th of October 2023, Israel launched an oppressive war on the Gaza Strip, creating unbearable conditions, that the UN International Court of Justice has declared it in its ruling of 26th January 2024 to be at least, plausibly a genocide (International Court of Justice, 2024). Part of this war has been a “total annihilation of education”, or what is so often described as a “scholasticide” (Scholars Against the War on Palestine, 2024, p. no pagination). In the context of this Israel’s ongoing war on the Gaza Strip, scholasticide “describes the systematic destruction of Palestinian education [including] the intentional destruction of cultural heritage […] killing, causing bodily or mental harm, incarcerating, or systematically harassing educators, students and administrators […] besieging, closing or obstructing access to educational institutions” (Desai, 2024, p. no pagination). According to Scholars Against the War on Palestine (2024), Israel bombed every university in the Gaza Strip, preventing more than 90,000 university students from studying for HE. But even when Israel’s war comes to end, students may not be able to return immediately to study because of displacement conditions, the loss of expertise, and since these universities were completely or partially destroyed. The process of healing and reconstruction for these universities and their educationalists would be challenging. It is hoped that some of the insights in this paper could be helpful in imagining the challenges that might be faced in any attempt to re-establish the system of HE in the Gaza Strip, and how to increase educationalists’ resilience in a context of continued instability and occupation. As it foregrounds the experiences of educationalists at Gaza’s universities in Israel’s 2014 war on the Gaza Strip, this article is also a valuable contribution to the documentation of the history of Gaza’s universities.

The paper in hand provides; firstly, the context for this study; secondly, the research methodology, including theoretical and conceptual insights; thirdly, the research findings; and finally, a conclusion which summarizes key take aways from findings, as well as outlining some implications of this research for the reconstruction of Gaza’s universities, after the end of Israel’s ongoing war on the Gaza Strip.

## The context

Education is a crucial component in any process of reconciliation, reconstruction (Leach & Dunne, 2007) and development (Allsop & Brock, 1993; Stewart, 2004). But Davies (2004) observes the relationship between education and conflict from two other perspectives, on the one hand, as “the ‘wicked’ end of change” since a change to one cannot happen without a change to the other and vice versa ( p. 21), and, on the other hand, as part of a non-linear (interactive) dynamic with other systems. While education could play a role in reconciliation and reconstruction, conversely, occupation and war circumstances affect education’s capacity to perform this role. For example, Wind (2024) explains “how Israeli universities actively sustain Israeli settler colonialism, military occupation, and apartheid, as well as their own complicity in the ongoing violation of Palestinian rights as recognized under international law” (p.10).

The universities in the Gaza Strip have been struggling under Israel’s occupation for decades. Restrictions on travel, investigations on the border, and a growing incapacity of neighboring Arab countries to host Palestinians, for study or work, has necessitated the emergence of Palestinian universities in the Gaza Strip. For example, the first Palestinian university in Gaza was established in 1978 under the Israeli occupation. Nonetheless the occupation did not contribute to it financially or academically (Al Anabtawi, 1986; Haq-Affiliate, 2005; Murray, 2004; Ramsden & Senker, 1993; Sullivan, 1988). In fact, it imposed several obstacles to its operation, including closures for a prolonged period, regular incursions and detentions of its staff and students, especially during the time of the first uprising (1987–1993) in the territory. Over decades, Gaza HE and its universities continued to be undermined by the Israeli occupation in various ways, including the siege and repeated wars, as we shall see shortly. But HE in the Gaza Strip has also been suffering from the consequences of competition between the agendas of two main Palestinian factions: Fatah (1959), and Hamas (1987). While Fatah favours the strategic approach of negotiating with Israel, Hamas has dismissed such a strategy as prolonged and pointless (Munayyer, 2011). The signing of the Oslo Agreement in 1993, allowed the Fatah-dominated Palestinian Liberation Organisation (PLO), which had previously been expelled to Tunis, to return and operate in Gaza forming the Palestinian National Authority (PNA) (Munayyer, 2011, p. 22). The relative improvement in conditions in the coastal enclave following the Oslo Accords, has led to an expansion of HE in the Gaza Strip, as many universities, and community colleges were established. But the Oslo Accords brought, as Jeresaty & Mezvinsky, (2004) state, “a flawed peace process that did not yield reconciliation but rather more violence and disillusionment” for the Palestinians (see also Jebril, 2006; Nicolai, 2007; Roy, 2007). Despite Israeli evacuation of the settlements in the Gaza Strip, with the implementation of the disengagement plan in 2005, Israel continued to control all aspects of life in the territory, including Gaza’s access to water, electricity, and exports and imports, as well as forcibly separating the Gaza Strip from the West Bank. (Jebril, 2024).

Perhaps this Palestinian disappointment in the Oslo Accords had *inter alia* given Hamas the legitimacy it needed to win the Palestinian elections in 2006. Hamas was democratically elected by the Palestinian people to act as a government, but this has driven the Gaza Strip, a stronghold of Hamas, into crisis, especially after the government schism, which led to two ruling regimes, the Fatah-dominated PNA in Ramallah (the West Bank), and Hamas in the Gaza Strip. Sanctions were imposed on the Gaza Strip, by Israel, the PNA and the International community. Israel imposed a tightened blockade/siege on the Gaza Strip by air, land, and sea. This siege had devastating consequences to HE in the Gaza Strip, as well as affecting faculty and students. For example, in Jebril (2018, 2024), I explain that restrictions on Palestinian mobility limited Gaza universities’ capacities for academic exchanges and affected Gaza’s development through lack of expertise, library resources, and technical and laboratory equipment. Teaching and learning at Gaza’s universities, was affected by a power cut of 6–12 h on daily basis, as academic staff and students struggled to do their academic tasks and responsibilities at home, due to the lack of working light and internet connection. The unpredictability of when this power cut may happen, also affected universities’ capacities to plan for events such as seminars and conferences or to operate essential online services such as admission platforms. The frequent shortages of fuel for transportation and to use for alternative power generators, also reflected on Gaza’s universities with disruptions. Worsening unemployment resulting from the occupation and siege, has frustrated students from the possibility of getting a job after they graduate, making them increasingly inclined to using surface learning approaches. In turn, all these challenges have reflected on class dynamics, encouraging an instrumentalist form of teaching, learning and governance. Increased poverty affected Gaza faculty and students’ health. It also limited families’ abilities to educate all their children, often choosing to give priority to males over females. Since 2008, Israel has further launched five large-scale wars (in 2008; 2012; 2014; 2021; and 2023-present). Two of these wars (2012, 2014) took place during the period of this research. Repeated wars have driven the Gaza Strip into further humanitarian and economic catastrophe affecting Gaza’s universities and their educationalists both physically and psychologically, as we shall see shortly. (Jebril, 2018, 2024).

This paper reports on Israel’s 2014 war on the Gaza Strip (8 July-26 August 2014), which compared to previous wars, was “unprecedented on many levels” (Shehadeh, 2015, p.278). The 2014 war left “over two thousand Palestinians dead, mostly civilians, over eleven thousand injured, and whole-scale destruction of neighborhoods, and infrastructure” (Shehadeh, 2015, p. 284). Monitor (2015) states that “for 50 days, more than 6,000 air strikes, 14,500 tanks shells and 45,000 artillery shells were fired on Gaza as Israel decimated the Palestinian enclave” (no pagination). The destruction hit even “United Nations personnel, premises and operations”, including UNRWA schools, which were used as family shelters during the war (United Nations, 2015, p. 1). Hubbard (2014) reports that “life in these shelters [was] full of deprivation and discomfort” (no pagination). Filiu (2014), explains that the 2014 Israeli war on the Gaza Strip “has left approximately 108,000 Palestinians homeless as a result of the total or partial destruction of some 18,000 dwellings” (p. 58). Israel also followed a “policy of collective sleep deprivation across all of the Gaza Strip [exposing people to] noxious sensory stimulations, across various modalities, including visual (illumination flares), auditory (deafening explosions), and tactile (ground-shaking explosions that rattle even the sturdiest of buildings)” (Shehadeh, 2015, p.286). As Milton et al. (2021) states, “whilst not unique in all aspects, occupation and blockade entails that Gaza constitutes an atypical conflict context and an extreme example of isolation during conflict with far-reaching consequences for higher education” (p. 1028). But there is hardly any sociological research on how Israel’s 2014 war on the Gaza Strip has impacted its educationalists.

## The research study

This paper draws on 36 in-depth semi-structured interviews, with educationalists (15 academic staff and 21 students) from the Faculties of Education at two of Gaza’s universities. It is based on an exploratory and inductive qualitative study which I conducted for my PhD degree at the University of Cambridge (2012–2017). Most of the interviews took place in the last quarter of 2014 (shortly after the war has ended), with a few extending into the early first quarter of 2015. Due to access difficulties, the interviews were arranged via Skype and phone/mobile from Cambridge. Prior to conducting the interviews, I reviewed the available interdisciplinary literature, as sociological writings on HE in Gaza were almost non-existent. I also reviewed the academic participant CVs, and created a chart which mapped their experiences across historical and political events in Gaza, past and present. Purposeful sampling was used to select lecturers while the convenience (snowballing) method was used to select students. As a Palestinian from the Gaza Strip, I addressed my insider–outsider positionality through, designing a self-interview protocol to be aware of my biases and assumptions prior to the fieldwork. This exercise also helped me to take a distance when listening to my participants’ experiences, as well as be attentive to any surprising thoughts or reflections in their sharing. What I learnt from this exercise, however, was how much we had in common, thus emphasizing that the difficulty in coping with the post-war experience, is not an individual sensitivity issue, but rather a joint struggle for the academic community in the Gaza Strip. I also designed a power-analysis sheet to anticipate interviewer-interviewee’s dynamics of communication, an interview guide, and a digital memo aide on Scrivener software (Jebril, 2021a, 2021b).

Informed by the two main principles of Goodhand (2000) regarding research in conflict areas, namely, doing no harm and doing some good, I monitored ethical and security issues before, during, and after the study. For example, the interview questions were designed to facilitate access to the required data only. Informed consent was obtained orally for a culturally sensitive practice. As Ford et al. (2009) indicate, “the process of obtaining informed consent must be sensitive to the norms, customs and sensitivities of the local environment” (p.5). During the interview, I remained attentive to any explicit or implicit expression of constraint or distress by the participants when talking about their experiences (Goodhand, 2000, p. 14). Both the anonymity and confidentiality of participants were protected at all stages of the research. I designed a safe data storage system (see Jebril, 2018). This included pseudonyms for both institutions and their educationalists. In this article, I use the pseudonyms of the two sampled Gaza universities as surnames: UA and UB, and I refer to academic staff as ‘Mr/Ms [name] UA/UB’, and students as ‘[name] UA/UB’. To clarify, Ms Randa UA is a female lecturer whose first name is Randa and who works at the UA university; Waleed UB is a male student whose first name is Waleed, and studies at the UB university.

The interviews were analysed thematically. I firstly coded the interviews using the analysis software MAXQDA, and then, using the cut and paste digital function, arranged the coded data into theme categories and sub-categories. The analysis was conducted creatively, by interpreting data in relation to each other, and to theory and the literature.

## Theoretical insights

The inductive study has benefited from conceptual insights, namely from Freire’s *Pedagogy of the Oppressed*, and Bourdieu’s symbolic violence retrospectively. The “intimate connections between life and philosophy” in Freire’s work makes it relevant “to the contemporary analysis of education and culture” including this research (Irwin, 2012, pp.1 & 8). In this paper, I use several Freirean concepts such as ‘oppression’, ‘fear’, ‘fatalism’, ‘horizontal violence’, duality’, ‘manipulation’, and ‘dehumanization’, to make sense of the data (Freire, 1996, pp. 37, 30, 43, 44, 30, 128, 26). For definitions of these concepts, see (Jebril, 2018, 2021a). Freire’s work was “originated in Third World countries”, but oppression in the Gaza Strip has a different texture and is influenced by its national context and Arab culture (McLaren & Leonard, 1993, p.5). Freire’s book, took “cue from the [specific] situation in which it [found] itself” (Irwin, 2012, p.11), which is different from this time and the Palestinian context. I attempted a “cultural synthesis” between a number of his concepts (some of which have been rarely used, if at all, in the literature on HE), and my empirical data as relevant, applied them flexibly, and whenever possible problematized them (for example, in relation to fatalism) (Freire, 1996, p. 161).

The paper also draws on Bourdieu’s notion of ‘symbolic violence’. For Bourdieu, symbolic violence refers to the distortion of an individual’s autonomy that restricts the person from acting in his/her own interest, by virtue of others’ capacity (symbolic power), and authority (symbolic capital) to impose symbolic meanings on them. These symbolic meanings act as “dispositions” that cause the individual to fear the anticipated the consequences of his/her actions, and to “comply” with the oppressor (Bourdieu in: Swartz, 2013, p.89, 39). Freire’s and Bourdieu’s selected concepts together provided an insight into the nuances of the Palestinian HE experiences at Gaza’s universities on a deeper level, allowing the analysis to move from the concrete to the abstract.

Freire’s and Bourdieu’s works are interrelated as they are both focused on the analysis of the social world, and concerned with the oppressive experience, but there are also fundamental differences between both. While Freire is optimistic about the possibility of transforming from an oppressive situation through critical pedagogy, Bourdieu perceives this as an “intellectualist illusion […]. It misunderstands the depth of oppression, for it […] obscure[s] its class underpinnings. Freire might retort that Bourdieu is focused on the transmission of the dominant culture and cannot see beyond a banking model of education” (Burawoy, 2012, p. 112).

In fact, both Freire’s and Bourdieu’s perspectives on oppression could be relevant to this research on the Gaza Strip. That said, HE in Gaza has a transformative potential, that could be useful to empowering the society, and thus supporting its efforts towards resilience at times of war. Nonetheless, Gaza’s universities could be sometimes facilitating societal oppression, as they suffer from a state of reproduction due to decades of occupation which put constraints over their essential funding sources, as well as limiting Gaza universities' exposure to academic and cultural development opportunities. This has resulted, *inter alia*, in these universities’ conservativism to traditional practices of teaching, and governance, undermining HE’s capacity for transformation. That said, it was important then to “adopt a broader theoretical canvas […to] grasp the ways in which education becomes a terrain of struggle that fosters both […] change and […] reproduction” (ibid., p. 118). Conversely, the author, combining these theorists work, has created an opportunity for these theorists’ concepts to be in dialogue with each other, and with the author, on the data from the Gaza Strip. This article provides only examples of this, but more details can be found in (Jebril, 2018). In this paper, I have applied Freire’s and Bourdieu’s concepts tentatively and sporadically, only, as landmarks of oppression. I synergize insights from both Freire’s and Bourdieu’s by using their concepts simultaneously in interpreting the data, as suitable to each situation.

Raewyn Connell (2007) states that “mainstream sociology turns out to be an ethno-sociology of metropolitan society” (p.226). However, as Bourdieu (1993) argues, “one cannot grasp […] the social world unless one becomes immersed in the specificity of […the] empirical reality” (p. 271). Using a social constructionist approach, I constructed the findings below by synergising insights from the interview data, theory, and the literature, although primacy in the analysis was given for empirical data from the Gaza Strip.

## Findings: the war experience for educationalists at Gaza’s universities

In this section, I consider; firstly, the experiences during Israel’s 2014 war on Gaza, and how the war circumstances increased the vulnerability of educationalists at Gaza’s universities, despite their efforts for resilience; secondly, the extended impact on lecturers, students, and their universities, even after the war came to end; and finally, a dilemma for Gaza’s universities, which undermines their academic work and development.

### During the War: Participants reflections between vulnerability and (the illusion of) resilience?

Understanding the present HE experience at Gaza’s universities without understanding the war experience does not make sense, as wars in Gaza are not coincidental. The Israel’s 2014 assault on the Gaza Strip was devastating to the Gaza society and its universities. According to B. B. C. News (2014), “17,200 homes [were] destroyed or severely damaged by Israeli attacks” (no pagination). Solberg (2014), describes that “a broken metal wheelchair stood in the ruins, crushed by the massive force of an explosion, its owner dead or in hospital […and that] elsewhere in the rubble were traces of the lives” (p.389). During the war, educationalists at Gaza’s universities perceived themselves as if they were in a horror ‘action movie’. Mr Abdel Rahman UB said: ‘*Imagine that with the bombing of […this] tower, […] an iron leaf door […] got to our house*’; and Mr Majid UB stated:

I heard the name of my family mentioned among [the houses which were] bombarded. I rushed [to the site] and found the F16 has destroyed the home of my cousins. These young men were from outside Gaza, and they were coming only to study at the university.

In the interview, Majid explained that these students were brothers, and that he thought it was their destiny that one of them survived from under the rubble, while the other have become ‘martyr’. This incident was shocking and saddening for Mr Majid UB. As for the student who survived this horrific experience, he had yet to endure the additional trauma of mourning his killed brother.

During Israel’s 2008–9 war on the Gaza Strip, Amditis (2012) states that Israel used “white phosphorous (WP) shells, not only against Hamas militants, but also against the civilian population of Gaza” (p. 15). Mojabi et al. (2010) add that “using [WP in 2008–9 operation] also caused […] environmental pollution” (p.125). Similarly, in Israel’s 2014 war on the Gaza Strip, Alaa UB was affected by the war chemicals. She had a miscarriage and had an operation during the war. She said: ‘*Alhamdullillah, may Allah compensate us’*. In the interview, fatalism seems to have helped this student to come to terms with her loss, as she believed that, after all, it was her destiny to have a miscarriage. Undergoing an operation in war time in Gaza, was a dehumanizing experience for Alaa. Hospitals and health facilities were also targeted by Israeli air strikes (Solberg, 2014). Also, Shehadeh (2015) explains that Gazans were repeatedly exposed to “gruesome scenes of dead bodies, and body parts, lying in pools of blood after being torn apart by high-intensity explosives” (p.284). Mr Omar UB reported: ‘*The Israelis hit the car with a rocket […] I had blood, pieces of clothes, and some flesh on me from the person who was killed*’. Mr Abdel Rahman UB, feared these war conditions would cause epidemics in Gaza.

The 2014 Israeli war on the Gaza Strip had a significant psychological impact on educationalists at Gaza’s universities. Students had several fears, but mostly talked about the fear from losing a part of their body, a member of their family, or their house. At least three students feared becoming disabled. Ferial UA mentioned having ‘*[horrifying] nightmares of [their] bod[ies] dismembered and [their body] parts scattered all around the road […and that this] made [them] feel distracted from their studies*’. Tamara UB had combined fears. Tamara said:

I was afraid of losing one of my family members. I was afraid of being homeless and I was afraid to be disabled for the rest of my life […]. Sometimes, I thought that I wish I could die. I wish that there would be a bomb that would kill us all, all the family, once and together forever […], because […] deep down you still know that this is not the last war [and that] next time, you might lose something important to you.

Tamara felt fear and anxiety towards what might happen if a bomb or a rocket was dropped on their home. This female student seemed hyper vigilant as she expected that Israel would continue to launch wars on the Gaza Strip. Tamara indulged herself in imagining the current and future consequences for that pessimistically. She feared that all possibilities would be tragic, for example, causing her disability or destroying her home, or killing one of her family members. Tamara attempted to exercise her agency by wishing what kind of death she would ideally hope for, which is to die with her family, ‘*once and together*’. Like Tamara, at least four other students feared, remaining alone if they lost their dear ones to the war.

Compared to students, however, the academic staff seemed reserved about expressing their fears. Ms Amna UA, Mr Omar UB, and Mr Mehdi UA pointed out their concern about their children, and Mr Abdel Rahman UB stated: “*I was thinking about where [the Palestinians,] are going. I cannot see any future for us. Hope? I cannot have any*”. Mr Abdel Rahman UB who is an academic staff in his late sixties, spent most of his life in the Gaza Strip, witnessing repeated Israeli attacks on the Gaza Strip. Abdel Rahman seems to have internalized feelings of vulnerability and pessimism about the future in a fatalistic way. Ms Randa UA, another academic, said:

There is no one who has not felt afraid in the war [...]. However, no one wanted to leave his house. No one [...]. We have not left except for five days only. We were obliged to leave because everyone around us left, and [...] we had bombs dropped in our house, destroying part of it [...]. I was as if seeing a flash of the past photos of the 1948 [....]. They said they wanted to take our house. Can you imagine what a suffering this is [....]? Imagine that in a moment everything, all your work, and all your life comes in front of you as nothing! [...]. This has strengthened in me that our life battle is not with anyone else; it is with the occupation.

For Ms Randa UA to be forced to leave her home was dehumanizing and instilled in her feelings of fear, helplessness and insecurity. Randa attempted to justify why she left, by explaining that she was ‘obliged’ to leave. This suggests that Randa was aware that as an academic staff, she was perceived as a role model for her students and her community. She felt defensive, as she was not able to show resilience by staying in her home, when bombs were dropping on it, and her surrounding neighbours had left. Ms Randa UA’s identification of her situation with the *Al Nakbah* exodus caused her to view her own oppression from a historical perspective, as a fate for Palestinians. Paradoxically, fatalism here has endowed Randa with a sense of resilience as she engaged in a ‘*life battle*’ against the occupation. Like Randa, most participants had evacuated their homes or hosted others who had. Returning to his house after the Palestinian-Israeli truce, Khaled UB felt exhilarated. He said: ‘*I went kissing the walls of our house. Yes, I kissed the ground. I kissed my desk […]. Alhamdullillah, it was not damaged’.*

Israel’s 2014 war on the Gaza Strip has destroyed not only lives and homes, but also important occasions. Halima was a newly married bride, but she inhaled smoke and gunpowder, as rockets were falling in her neighbourhood area. Her case implies dehumanization. To express this metaphorically, stability was replaced by instability, joy by fear, celebratory fireworks by killing rockets, and the scent of perfume by the smell of gunpowder for this student bride. The war also took place during part of the Holy Month of Ramadan when participants were fasting. Although this, as Khaled UB stated, ‘*comfort[ed…them] for their losses’*, it was hard because of the dehumanizing conditions, of increased periods of water and power cuts. According to UNCTAD (2015), the Israeli “operation resulted in severe damage to Gaza’s water and sanitation structure […and] damaged Gaza’s single power generation station” (p.12). On this, Ahmed UA commented, ‘*Imagine yourself living in [hot] summer without any electricity at all, without even cold water to drink, without a fan to have some fresh air […]. 2014 [was] the worst year ever’*.

Under difficult circumstances of the war, religion and a sense of fatalism seems to have provided people with psychological assurance. Mr Abdel Rahman UB, for example, mentioned: ‘*During the war, one used to say lā ʾilāha ʾillā-llāh, muḥammadur-rasūlu-llāh (the testimonial word) every ten minutes. Also, one had to accept whatever happened*’. At least three students also perceived their lives as futile as they felt alienated during these 50 days war on the Gaza Strip. Nawal UA stated:

The lesson I learnt from the war is that everything in life is trivial, because everything could vanish in a moment. Everything is in the hand of our God. We could die in a moment; we could lose anything in a moment [...]. The only thing we should work on in our life is that to work for our afterlife [...]. I tried to write on Facebook statements that would increase the morale of my friends. To remind them, for instance, of the Final Day, and to tell them: ‘*As you might be meeting Allah, go and read some pages of Quran or pray [...]*’. So, I was trying to distract myself by these things, so I do not feel afraid.

Similarly, Khadra UA explained:

Everything we have done in our life is in vain […]. What did we do for our afterlife? I started to message my friends […]. Yeah, we’ve worked, we’ve done so and so, but what have we done for our afterlife?

Nawal’s and Khadra’s war experiences made them view life as futile. As they felt helpless, they became inclined to religiosity in a fatalistic way as both looked to the afterlife as a solution to the war situation. As Abū ʻAmr (1994) indicates, “the majority of the Palestinian people are Muslims, and Islam plays a basic role in Palestinian society” (p. xvi), so inviting Muslim colleagues to read the Quran or to pray is a socially acceptable/favourable practice in the Gaza Strip. But Nawal, and Khadra regretted investing efforts in their everyday life. As Ali and Al-Owaihan (2008) explain, “work in Islam […] is situated in the core of the faith and is considered an integral part of life” (p.7). In that sense, Nawal’s and Khadra’s increased religiosity appears to be less of a nurturing of faith, and more of a coping mechanism. For Waleed UB and Khaled UB, however, the war motivated them to think of their agency and contribution. Although Waleed ‘*kept reading the Quran, and praying*’, he was simultaneously considering how he could help Palestinians in the future. Khaled UB, the second example, took his novels with him when he evacuated the house. Reading novels was how this student aimed to restore his humanity amid bombardment. Khaled said: ‘*I hope I could […] contribute in the future’.*

During the war, lecturers and students had either moved from their houses in search of shelter or hosted others from their loved ones who had to move. This resulted in a double-edged social atmosphere: for some, it created a supportive environment for people during the long days of Israeli bombardment, but for others it caused them inconvenience and increased the pressure on them. On the one hand, Lubna UB said: ‘*there wasn’t electricity all the time, so we were playing Shadah cards*’. The war circumstances have paradoxically provided Lubna with an opportunity for family interaction. According to Hobfoll et al. (2011), “having greater social support [was] associated with being in the relatively more favorable or improving trajectories” towards resilience (p.1406). On the other hand, Ahmed UA explained:

I was talking with a friend who had 100 people in his house during the 50 days […]. He was telling me how difficult it was for them to feed 100 mouths, to cope with 100 in one house with this bombardment, with everything.

In Gaza, it is a social taboo to refuse one’s relatives if they come for refuge in one’s house, especially if they are from one’s immediate family. Consequently, Ahmed’s family ended up hosting many of their relatives. Ahmed UA explained that the pressure comes not only from being deprived of one’s private space, but also from struggling to be hospitable in these difficult circumstances. Similarly, Mr Ashraf UB described: “*Every day, I would need a packet of bread, but it was difficult for me to provide this*”. Almaney (1981) points out that in Arab culture, “a guest is considered almost a sacred trust” (p. 12). Also, “public face, and social approval are of paramount importance and moral worth is largely judged by others” (Sobh & Belk, 2011, p. 323). Ashraf’s social dispositions made him want to present a good image of himself as a host. In the interview, Ashraf explained that this put him under financial pressure and made him risk his own life by going to shop in places of bombardment. The conscious or unconscious calculation of the consequences that controls people’s actions is what Bourdieu calls symbolic violence. In the interview, Ashraf also said that he strove to provide hospitable chicken meals for his sisters’ families who took refuge in his house. He was keen not to embarrass them in front of their husbands, and to give a good impression of their family of origin’s socio-economic status. Ashraf feared horizontal violence from within his family. This thinking controlled his actions and placed pressure on him by way of symbolic violence as he feared the consequences of not being hospitable.

Academic staff and students’ earlier war experiences constituted informal education which influenced their dispositions, and consequent reactions to the dehumanizing situation they have encountered during Israel’s 2014 war on the Gaza Strip, strengthening their resilience. A few educationalists reported that the war lost its symbolic power over them, as they became used to these circumstances. For example, Talal UA explained: *‘It makes a difference whether this is the first, second, or third war’*, and Mr Omar UB stated:

From the start of our lives, we are living in a war atmosphere. If someone dies, it means that his lifetime has simply come to an end […]. 90 per cent of the people did not even bother that there was a war going on […]. You would find the aircraft firing, but people […] won’t hide.

Most interviewees expressed their fatalistic views and acceptance of predestination regarding their own death if it happens, although this was particularly noticeable in the academic staff, namely, those from the older generation such as Mr Omar UB, who seemed used to repeated experiences of loss and suffering. According to Ragab (1980), “such a *post facto* acceptance of a mishap […is beneficial] for the psychological adjustment of the individual […as] this should help clear the mind for constructive action” (p.515). There is a difference between fatalism, and desensitization as the latter may be “associated with low empathy and proviolence attitudes” (Funk et al., 2004, p. 33). On the contrary, Israel’s 2014 war on the Gaza Strip seems to have increased Palestinians’ empathy as it improved their understanding of loss and suffering in their community. They resorted to fatalism to console each other, by accepting these as a pre-destination for Palestinians.

Hobfoll et al.'s (2011) argue that HE was found to be “associated with more favourable outcomes following trauma exposure” (p.1401). Mr Majid UB, who holds a degree in psychology, seemed to draw on repeated war experiences to support his children under such circumstances. The academic explained: ‘*I became more experienced in protective measures’.* Majid developed a technique to help his children. He said:

I succeeded in making our children […] close their ears and stretch on the floor whenever they hear the rocket sounds, and then to clap, dance, and sing when this is over […]. This is for them to feel safe […] and to discharge their negative emotions.

Majid trained his children to manipulate their fears in order to feel safe. But that his children did not project their feelings of fear, should not mean that these children did not actually feel afraid. His children’s rejoicing should not be mistaken for resilience, it is a temporary coping a mechanism. A specialization in psychology also gave Mr Majid UB, and a few other academics, the symbolic capital to support others, which was empowering for both.

Educationalists seem to have become more accepting of their fate when they compared their suffering to that of others. Below is my conversation with Waleed UB:

**M:** Did you have to move out of your house during the war?

**Waleed UB:** Twice.

**M:** How did you feel about that?

**Waleed UB:** I did not care.

**M:** Why not?

**Waleed UB:** Because I was seeing people suffering more than me, so I thought there was no comparison.

Also, Ms Jamila UA who lived for some time in Syria, stated:

I told the students that the Gaza war is much less intense than that in Syria. Here, you would be shot dead by a bullet or killed by a bomb, but, in Syria, you would be slaughtered [….]. Also, in the war on Gaza, we are confronting […], the Israeli [occupation], but in Syria, people are killing others from their own community.

Because Jamila had spent all her life in conflict areas, she seems to have internalized oppression as a fate, and as natural. In the interview, Jamila explained that this comparison between Gaza and Syria helped her to empower her students. That said, a “sense of relative advantage limits the sense of deprivation, and thus the oppressive system as it is felt or as it envelops group life is used as a positive frame of reference” (Wolf, 1986, p. 222). Another academic, Ms Etaf UB, indicated how during the war, her relationship with students proved empowering to both:

I was surprised that I have written a research paper, got it reviewed, and published it. […]. My master’s students used to call me about […] their theses […]. Because, I have seen my students working, I told myself that I should work too […]. As lecturers, we are [also] obliged to continue despite the circumstances, because this is our work, and this is our message.

At least three other academics, Mr Zeyad UB, Mr Abdel Rahman UB, and Ms Randa UA perceived themselves to be on a similar mission to educate the new Palestinian generation. Their religious and social dispositions in relation to their academic roles have endowed them with a sense of responsibility.

### After the war: a new post-war life demarcated by violence

After the attack on Gaza was halted, universities resumed working, but most participants struggled to return to normal life. Ahmed UA stated:

It was as if we were not in this city and now, we are here again. I felt that we were somewhere else, if I may say, in hell, and then after more than one month, we get up with the sunshine and go to the university […]! I think it is never going to be normal.

Ahmed’s perception was clouded by memories of fear and dehumanization that impacted on his ability to enjoy the sunshine. Mr Hassan UA also commented: ‘*Everything is ruined […]. This affects me because I am not living on a star, or another globe*’. According to UNCTAD (2015), “the United Nations Special Coordinator for the Middle East Peace Process, during a visit to Gaza in April 2015, […observed that …] as shocking as the devastation of the buildings might be, ‘the devastation of people’s livelihoods is 10 times more shocking’” (p.9). The destructions all around Gaza acted as a constant reminder of the dehumanizing war experience, while simultaneously, creating a feeling of empathy among Mr Hassan UA and others from his community. Some buildings were levelled to the ground. The erasing of these was not only an act of physical violence, but also symbolic violence. Tamara UB’s quote illustrates this:

I do still have nightmares and every single time, I pass by the Italian tower they bombarded […], I can remember […] the sounds of explosions […]. I remember that night. It left a scar in my heart.

Israel’s bombardment of the Italian Tower that was there, have changed the landscape that Tamara used to pass by in her way back and forth from the school in which she used to train as a teacher. As a result, Tamara suffered internally, as this evoked difficult memories and nightmares for her. Tamara’s account is consistent with Audergon's (2004) argument that “the experiences of traumatized individuals include […] the violent reply and intrusion of events in flashbacks, nightmares, visceral experience of the events and body symptoms” (p.19). Displaced people in schools have also acted as reminders of that oppressive experience, emphasizing people’s dispositions of fear and vulnerability. That said, “over 500,000 Palestinians were displaced during the [Israeli] operation, with some 100,000 continuing to be displaced by mid-2015” (see UNCTAD, 2015, p.9). For example, Waleed UB stated: ‘*I can see the displaced people living in the schools […,] this affects me’*. Mr Hassan UA explained:

I noticed something about my students: a lot of them don’t smile […]. While I am driving, I am [also] watching the people who stand on both sides of the road [and wonder] why none of them smile or maybe laugh.

Hassan observed it to be an unusual thing that none of his students, or people on the road were smiling. Indicating that this was not the case before the war, suggests that Israel’s 2014 war on the Gaza Strip had a shared frustrating impact on the Gaza community, that continued to affect them even after the war ended. Samira UA explained that even after the war ended, she ‘*continued to have nightmares’*. Hence, construction should target not only the tip of the iceberg of destruction, namely, the physical (visible) damage, but also what is underneath it, that is, the invisible and symbolic impact. Mr Mehdi UA also indicated that the war impacted negatively ‘*on the youth in terms of their academic achievement and […] concentration*’. People wanted to desperately forget the past fears and memories of war. Halima UA stated: ‘*when I went to the university, everyone tried to avoid speaking about the war [….], they just wanted to end this memory*’. Similarly, Samah UA pointed out that ‘*all students refused to speak [about the war]*’. Educationalists tried to manipulate their memory, but deliberately repressing one’s memory in order to forget, rather than allowing the self to heal naturally, is a form of memory abuse that should not be mistaken for resilience (Ricoeur, 1999). According to Ricoeur (1999), “memories have not only to be understandable, they have to be acceptable” (p.7). Audergon (2004) explains that trauma in Gaza has become a “collective dynamic” (Audergon, 2004, p. 16). Mr Riyadh UA argued that ‘*all people have been traumatized to different degrees [….], but [they] have also coped with [this trauma] to different degrees*’; and Mr Mehdi UA said: The ‘*trauma will remain in [educationalists’] memories, in [their] consciousness, and it would affect [them] in all aspects of [their lives]*’.

### Gaza’s universities and challenges for development

In the context of the blockade in which “Gaza’s airspace, maritime space and land crossings” are controlled by Israel, Gaza HE institutions rely mainly on their human resources for development (UNCTAD, 2015, p.9). Lecturers and students, as professionals, are also necessary to the organization of a new Palestinian society, especially after this destruction. After the war, these human resources were themselves ‘*sinking in darkness*’ (Tamara UB). At least six UA participants reported the targeting of their university. Ms Amna UA stated: ‘*My office in the faculty was […] affected’.* Talal UA also reported: *‘The administration building was bombarded. There was rubble. The conference hall was also affected. Even the UB was affected as it had its windows broken’*. Ms Randa UA, an academic working in administrative position, gave a vivid description of her return to the university:

We […discussed] how we were going to deal with the situation […]. There is an educational mission that needs to go on. We need to educate our students, to rehabilitate them. But we cannot do that without rehabilitating ourselves first.

Academics felt the responsibility to support their students after the war ended. But like students, academics were affected by their war experiences, and so they were in need for support as well. In the interview, Ms Randa UA explained that the luxury of professional mental health support was not available so the only way for them was to adapt to the difficult conditions. As for Mr Zeyad UB, he said: ‘*We need to rehabilitate people because as we are the well-educated ones. We need to transcend ourselves to look after those who would follow us’*. The quote from Mr Zeyad UB has a paternalistic tone to it that subscribed to the traditional assumptions regarding old versus young in the Arab world. According to Barakat (1993), the hierarchal structure of the Arab family based on age “creates vertical rather than horizontal relationships between the young and the old. In such relationships, downward communication often takes the form of orders, instructions, warnings, threats, shaming and the like” (p. 106). In the traditional society of the Gaza Strip, this family hierarchal structure extends to the university as the lecturers, usually older in age than students, assume a parental/maternal supervisory role over students. Two academic participants, Ms Etaf UB and Ms Jamila UA, also did not share their true thoughts and feelings with their students. Etaf explained: ‘*Our role dictates that we tell people that there would be hope, life and future, but in fact we are deluding them and ourselves*’. The academic staff, including Ms Etaf UB, seemed to perceive it as a nationalistic duty to manipulate their voices and show resilience. Ms Jamila UA, as another example, stated:

I went to the university for a week, and it was an excellent opportunity for me […] to inject students with high morale [….]. I am a member of the academic staff. I should always have a high morale […]. I am afraid not to appear in the same image.

Jamila’s concerns about peoples’ expectations of her caused her fear and increased her anxiety. As we have seen above, in the examples of Jamila, Etaf, and Zeyad, even when, it came to empowerment, these academic staff assumed that they were the authority whose role involved ‘injecting’ students with a high morale, by “the gradual addition of declarative [empowerment] knowledge to [students as] an essentially blank state or tabula rasa” (Lawson, 1988, p.195)*.* They did not “listen to the [students], but instead plan[ned] to teach them how to ‘cast off the laziness which creates underdevelopment’” (Freire, 1996, p. 137). These academic staff tried to act as psychological role models for their students instead of negotiating with them how, in the aftermath of the war, the students might find courage within themselves and in their own way. But Academics’ feelings of responsibility towards their students, and their keenness to present themselves as models, seems to have increased not only academics’ anxiety, but also their resilience in relation to the war.

The return of educationalists at Gaza’s universities was fraught with conflicting expectations that fostered horizontal violence. Tamara UB argued:

I expected that the university would […] be dealing with us with some ease […]. The lecturers were demanding us to attend all the classes […]. Aren’t [they] human [s]? […]. One of the teachers gave us like 400 pages to study […]. But […] we were just out of a war, so [he] should [have been] careful.

This same semester that Tamara mentioned here was described by Ms Lamia UB, as the ‘*most difficult semester in [her] whole academic life*’ which spanned more than 20 years. Ms Lamia UB indicated that as lecturers, they ‘*started teaching, and then, [they] had to stop, then they got back, then [they] had to stop again as the war resumed and finally, they got back again*’ when the attack came to a halt. The post-war return to the university was dehumanizing for both students and the academic staff. Yet, Tamara UB was convinced that her lecturers were inhumane, and insensitive. It is of course understandable why Tamara UB, even though she was a first-class student, would need this ‘ease’ in the aftermath of the war experience, but lecturers’ strictness, despite the war circumstances, is also understandable. The disruption and the limited duration of the semester did not allow for the provision of these summer courses to be completed in accordance with high standards. At least three UB students did not have the time to revise for their examinations. Ms Lamia UB explained:

The university did not consider [us]. They wanted to hurry and open the new semester for registration, because the university has also financial and economic pressures […]. Consequently, […] it is for the lecturer then to see how to help the students.

From the quote above, not only students complained about how they were treated by the academic staff, but also the academic staff complained about how they were treated by their universities after the war ended, which Lamia perceived as not considering them. Gaza’s universities, despite their peculiar context, remain bound by the expectations placed on them to be “institutions of higher education […to be] devoted […] to universal learning” adhering to high standards (Kerr, 1990, p. 18). But academics felt they had the responsibility to support their students simultaneously as they offer them a high quality HE. Combining these together was an impossible task for the academic staff who had to speed up in the educational process because of financial considerations for their universities. This affected lecturers’ teaching approaches. Amira UB stated: ‘*during the truce, [students] went to their class, but [despite the sounds of explosions around], the lecturers did not stop explaining the course material*!’. UA students also expected their lecturers to deal in an understanding way after the war ended. Nonetheless, Dalal commented: *‘we thought the exams would be easy […]. But they were so hard [….]. Maybe the [lecturers] had the exams ready before the war’.* Samira UA also stated:

They promised us to take care of us in the marking process, but these promises were false […]. They should have been more humane with us [….]. Both of us have seen and lived in the same conditions [of war…]. There is a lecturer who […] failed half of the class and most of the students whom he failed have already had their homes destroyed or have been displaced from one place to another.

Students had to undergo an additional frustration because of the war, which was their failure in one or more of their university courses. Both Dalal and Samira feared their marks would not be good. These students’ over-concern about marks was because of the widespread unemployment and the fierce competition over jobs in the Gaza Strip. Contrary to students’ complaints, Ms Etaf UB stated: *‘after the war, my teaching strategy had to change. I am not able to ask [the students] for assignments’.* Ms Etaf UB adjusted her teaching in consideration of students’ situation. Other lecturers also argued that after the war, they tried ‘*not to fail anybody*’ (Ms Lamia UB), but they struggled on how to balance this support for students with achieving a high-quality educational system. The support offered for educationalists by their universities was not substantial. The challenges that both the UA and the UB had to face because of the Israel’s war on the Gaza Strip were greater than these universities’ capacities to cope. According to Mr Hassan UA, there were also students at these universities who had become orphans or widows, and this had ‘*caused social problems in education*’.

## Conclusion

All in all, participants’ reflections on their war experiences showed an unstable tension between the positions of fear and vulnerability and that of empowerment. Educationalists clung to any possible means to survive oppression. History, religion and psychological knowledge were helpful. Social traditions, social solidarity, earlier experiences of wars, technology, novels and academic work and interactions were also beneficial. Even fatalism was sometimes useful to foster resilience. But it remains highly contested whether these social and educational ‘antibiotics’ have created resilience or only an ‘illusion’ of resilience. So far, the data from this research on the war experience have shown that, students and the academic staff remain deeply torn between their actual vulnerability and their struggle for survival, resilience and symbolic power. Milton et al. (2021), in another article about protecting HE from attack in the Gaza Strip, argue that “Gazan students and academics express fatalism by word and resilience by deed” (p.1039), which potentially could be an interesting point for future research.

After the war, Gaza’s universities found themselves in a dilemma over how to manage reconstruction since their staff, students and facilities had been affected by the war. The result was the duality of these HE institutions, which acted as both a supporter and an oppressor of their academic staff and students as, at times, they prioritized financial considerations over the needs of lecturers and students and in the process dehumanized them. Ms Jamila UA said: ‘*If you have a burden and I have a burden, then who should comfort who*?’. One might argue, it would be better if all comforted each other. Although this might seem an ideal compromise, it is very difficult to put into action in such a complicated situation like that of the universities in the Gaza Strip. That said, “the scale of what needs to be done in order to reverse the process of ‘de-development', and increase the weight of construction in comparison to destruction at Gaza’s universities is huge” (Jebril, 2021a, p. 1001), but for any development to happen, supporting academic staff and students should be first on the agenda.

Insights from the experiences in Israel’s 2014 war on the Gaza Strip are likely to be relevant to Israel’s ongoing genocidal war on the Gaza Strip, supporting the process of reconstruction at Gaza’s universities (and other contexts of war and conflict, particularly those of a similar Arab and Muslim culture): Firstly, the research highlights the importance of developing of a strategy of compassion, one that balances Gaza’s universities need to proceed as soon as possible with ‘business as usual’, but simultaneously acknowledge the impact of the war on their academic and student community. Linked to that is the importance of providing professional counselling support, as well as considering the specificity of the Gaza Strip in any planning for teaching, and learning (e.g. curricula, examination, term intensity, and goals) to encourage a deep approach to learning that is global, but also home-grown and relevant; secondly, Gaza’s universities could play a significant role in empowering community solidarity at times of war and adversity (e.g. through organising programmes of community education in history, religion, psychology, and protective measures at times of war and crisis); thirdly, increasing societal and university resilience requires that Gaza’s universities expand their circles of support, for example, to work actively to, establish academic partnerships with international universities, and open venues for donors to engage in the process of reconstructing the universities in the Gaza Strip, as appropriate to these universities’ missions, so as to reduce their over-reliance on tuition fees which may undermine their capacities for innovation following the devastating war circumstances; fourthly, Gaza’s universities would benefit, from investing in technological learning, and technological storage systems, to provide remote spaces for the universities in the Gaza Strip, that can be an alternative to deal with access limitations or partial/total physical destruction of their campuses at times of war and crisis; fifthly, aesthetic considerations should be part of any reconstruction efforts, in order to build new constructive memories in the post-war Gaza Strip; and finally, it is recommended that future research may build on this research article, to examine how discourses, practices and structures might be developed in a highly constrained environment, and how would it be possible to create an agile HE system that could be sustainable in the Gaza Strip and other occupied territories, at times of war and crisis. Israel’s destruction of all Gaza’s universities seems like orders of magnitude more violent than the 2014 war, requiring unprecedented reconstruction efforts on the part of Palestinians and their international supporters. With global academic solidarity, Gaza HE can recover, and the universities in Gaza can be rebuilt again.

## References

[CR1] Abū ʻAmr, Z. (1994). *Islamic fundamentalism in the West Bank and Gaza: Muslim Brotherhood and Islamic jihad*. Indiana University Press.

[CR2] Al Haq-Affiliate. (2005). Palestinian education under Israeli occupation. In *International Law in the Shadow of Israeli Occupation*. Stockholm.

[CR3] Ali, A. J., & Al-Owaihan, A. (2008). Islamic work ethic: A critical review. *Cross Cultural Management,**15*(1), 5–19.

[CR4] Allsop, T., & Brock, C. (1993). *Key issues in educational development: Vol. 3(2)*. Triangle Books.

[CR5] Almaney, A. J. (1981). Cultural traits of the Arabs: Growing interest for international management. *Management International Review,**21*(3), 10–18.

[CR6] Amditis, J. S. (2012). *War crimes in Gaza*. http://www.academia.edu/download/30579726/War_Crimes_in_Gaza_-_Operation_Cast_Lead_in_International_Law.pdf. Accessed 12/08/2016.

[CR7] Anabtawi, S. N. (1986). *Palestinian higher education in the West Bank and Gaza*. Routledge and Kegan Paul.

[CR8] Audergon, A. (2004). Collective trauma: The nightmare of history. *Psychotherapy and Politics International,**2*(1), 16–31. 10.1002/ppi.67

[CR9] B. B. C. News. (2014). *Gaza crisis: Toll of operations in Gaza*. BBC News. http://www.bbc.co.uk/news/world-middle-east-28439404. Accessed 14/08/2016.

[CR10] Barakat, H. (1993). *The Arab world: Society, culture, and state*. Univ of California Press.

[CR11] Bourdieu, P. (1993). Concluding remarks: For a sociogenetic understanding of intellectual works. In *Bourdieu: Critical Perspectives / edited by Craig Calhoun, Edward LiPuma and Moishe Postone.* Polity Press.

[CR12] Bouris, D. (2015). The vicious cycle of building and destroying: The 2014 war on Gaza. *Mediterranean Politics,**20*(1), 111–117.

[CR13] Burawoy, M. (2012). Pedagogy of the oppressed: Freire meets Bourdieu. In M. Burawoy & K. Von Holdt (Eds.), *Conversations with Bourdieu: The Johannesburg moment* (pp. 103–118). Wits University Press.

[CR14] Connell, R. (2007). *Southern theory: The global dynamics of knowledge in social science*. Polity.

[CR15] Davies, L. (2004). *Education and conflict: Complexity and chaos*. RoutledgeFalmer.

[CR16] Desai, G. (2024). *The war in Gaza is wiping out Palestine’s education and knowledge systems*. The Conversation: https://theconversation.com/the-war-in-gaza-is-wiping-out-palestines-education-and-knowledgesystems-222055. Accessed 08/02/2024.

[CR17] Filiu, J.-P. (2014). The twelve wars on Gaza. *Journal of Palestine Studies,**44*(1), 52–60.

[CR18] Ford, N., Mills, E. J., Zachariah, R., & Upshur, R. (2009). Ethics of conducting research in conflict settings. Conflict and Health, 3(1), 7 19591691 10.1186/1752-1505-3-7 PMC2717053

[CR19] Freire, P. (1996). *Pedagogy of the oppressed* (Rev. ed.). Penguin Books.

[CR20] Funk, J. B., Baldacci, H. B., Pasold, T., & Baumgardner, J. (2004). Violence exposure in real-life, video games, television, movies, and the Internet: Is there desensitization? *Journal of Adolescence,**27*(1), 23–39. 15013258 10.1016/j.adolescence.2003.10.005

[CR21] Goodhand, J. (2000). Research in conflict zones: Ethics and accountability. *Forced Migration Review,**8*(4), 12–16.

[CR22] Hobfoll, S. E., Mancini, A. D., Hall, B. J., Canetti, D., & Bonanno, G. A. (2011). The limits of resilience: Distress following chronic political violence among Palestinians. *Social Science & Medicine,**72*(8), 1400–1408. 21440348 10.1016/j.socscimed.2011.02.022 PMC3085932

[CR23] Hubbard, B. (2014, July 31). *School shelters in Gaza offer limited safety, and even less comfort*. The New York Times.

[CR24] International Court of Justice. (2024, January). *Summary of the Order of 26 January 2024*. https://www.icj-cij.org/node/203454. Accessed 18/06/24.

[CR25] Irwin, J. (2012). *Paulo Freire’s philosophy of education: Origins, developments, impacts and legacies*. Bloomsbury Publishing.

[CR26] Jebril, M. (2021). Between construction and destruction: The experience of educationalists at Gaza’s universities. *Compare: A Journal of Comparative and International Education,**53*(6), 986–1004.

[CR27] Jebril, M. (2021b). The Arab Spring: New campus dynamics at Gaza’s universities? *International Studies in Sociology of Education,**32*(3), 675–693.

[CR28] Jebril, M. (2006). *Reflections on higher education in Palestine: Barriers to academia and intellectual dialogue in the Palestinian-Israeli context* [Unpublished MSc Dissertation]. Oxford University.

[CR29] Jebril, M. (2018). *Academic life under occupation: The impact on educationalists at Gaza’s Universities* [PhD Thesis]. University of Cambridge.

[CR30] Jebril, M. (2024). *The Role of World Universities at Times of War and Crisis: Widening Participation and the Reconstruction of Higher Education in the Gaza Strip (Occupied Palestinian Territories)*. Cambridge centre for business research. https://www.jbs.cam.ac.uk/wp-content/uploads/2024/08/cbr-special-report-role-of-world-universities-at-times-of-war-and-crisis.pdf. Accessed 22/08/2024.

[CR31] Jeresaty, J. E., & Mezvinsky, N. (2004). The oslo peace Accords: A flawed peace process. Yayınlanmamış Yüksek Lisans Tezi: Central Connecticut State University, New Britain, Connecticut.

[CR32] Kerr, C. (1990). The internationalisation of learning and the nationalisation of the purposes of higher education: Two ‘laws of motion’ in conflict? *European Journal of Education,**25*(1), 5–22.

[CR33] Lawson, A. E. (1988). The acquisition of biological knowledge during childhood: Cognitive conflict or tabula rasa? *Journal of Research in Science Teaching,**25*(3), 185–199.

[CR34] Leach, F. E., & Dunne, M. (2007). *Education, conflict and reconciliation: International perspectives*. Lang.

[CR35] McLaren, P., & Leonard, P. (1993). *Paulo Freire: A critical encounter*. Routledge.

[CR36] Milton, S., Elkahlout, G., & Barakat, S. (2021). Protecting higher education from attack in the Gaza Strip. *Compare: A Journal of Comparative and International Education,**53*(6), 1024–1042.

[CR37] Mojabi, S. M., Ghourchi, M., & Feizi, F. (2010). Adverse consequences of conflicts and wars on environment and public health. *2010 International Conference on Environmental Engineering and Applications*, 125–129. http://ieeexplore.ieee.org/xpls/abs_all.jsp?arnumber=5596109. Accessed 12/08/2016.

[CR38] Monitor, E.-M. (2015). *Documentary film: Gaza, human shields*. Euro-Mediterranean. http://www.euromedmonitor.org/en/article/898. Accessed 02\08\2016.

[CR39] Munayyer, Y. (2011). Prospects for Palestinian unity after the Arab spring. *Insight Turkey,**13*(3), 21–31.

[CR40] Murray, H. (2004). *Barriers to Education*. Adalah’s Newsletter.

[CR41] Nicolai, S. (2007). *Fragmented foundations: Education and chronic crisis in the occupied Palestinian territory*. UNESCO International Institute for Educational Planning. http://lllp.iugaza.edu.ps/Files_Uploads/634714341026027290.pdf. Accessed 14/04/2014.

[CR42] Ragab, I. A. (1980). Islam and development. *World Development,**8*(7), 513–521.

[CR43] Ramsden, S., & Senker, C. (1993). *Learning the hard way: Palestinian education in the West Bank, Gaza Strip and Israel: Report from the 1993 WUS (UK) Study Tour*. Wus.

[CR44] Ricoeur, P. (1999). Memory and forgetting. In *Questioning ethics: Contemporary debates in philosophy* (pp. 5–11). Routledge. https://books.google.co.uk/books?hl=en&lr=&id=DiBF18LvcSoC&oi=fnd&pg=PA5&dq=memory+and+foregetting+&ots=SO01ByBE0S&sig=_XUCKxVeX1Gj_s8ubk-4q_d4oNw. Accessed 11/08/2016.

[CR45] Roy, S. (2007). *Failing peace: Gaza and the Palestinian-Israeli conflict*. Pluto.

[CR46] Scholars Against the War on Palestine. (2024, February). *Tool KIT: International Actions Against Scholasticide*. https://scholarsagainstwar.org/toolkit/. Accessed 24/05/24.

[CR47] Shehadeh, S. (2015). The 2014 war on Gaza: Engineering trauma and mass torture to break Palestinian resilience. *International Journal of Applied Psychoanalytic Studies,**12*(3), 278–294.

[CR48] Sobh, R., & Belk, R. W. (2011). Privacy and gendered spaces in Arab Gulf homes. *Home Cultures,**8*(3), 317–340.

[CR49] Solberg, K. (2014). Gaza’s health and humanitarian crisis. *The Lancet,**384*(9941), 389–390. 10.1016/s0140-6736(14)61125-9 25093221

[CR50] Stewart, F. (2004). Development and security. *Conflict, Security & Development,**4*(3), 261–288.

[CR51] Sullivan, A. T. (1988). *Palestinian universities under occupation* (Vol. 11). American University in Cairo Press.

[CR52] Swartz, D. L. (2013). *Symbolic power, politics, and intellectuals: The political sociology of Pierre Bourdieu*. University of Chicago Press.

[CR53] UNCTAD. (2015). *Report on UNCTAD assistance to the Palestinian people: Developments in the economy of the Occupied Palestinian Territory—Tdb62d3_en.pdf*. http://unctad.org/en/PublicationsLibrary/tdb62d3_en.pdf. Accessed 14/06/2016.

[CR54] United Nations. (2015). *Letter dated 27 April 2015 from the secretary- general addressed to the president of the Security Council*. http://blog.unwatch.org/wp-content/uploads/Board-of-Inquiry.pdf. Accessed 02/08/2016.

[CR55] Wind, M. (2024). *Towers of Iv**ory and Stee**l: How Israeli Universities Deny Palestinian Freedom*. Verso Books.

[CR56] Wolf, C. (1986). Legitimation of oppression: Response and reflexivity. *Symbolic Interaction,**9*(2), 217–234.

